# Supplementary material for: Carrageenans and the Carrageenan-Echinochrome Complex as Anti-SARS-CoV-2 Agents
Source: Int J Mol Sci. 2025 Jun 26;26(13):6175. doi: 10.3390/ijms26136175 (PMC12249508; doi:10.3390/ijms26136175)
Supplement: Supplementary file 1 [file ijms-26-06175-s001.zip › ijms-3700241-supplementary.pdf]

Supplementary material

Carrageenans and the carrageenan-echinochrome complex as anti-SARS-CoV-2 agents

Natalya V. Krylova[1,*], Anna O. Kravchenko[2], Galina N. Likhatskaya[2], Olga V. Iunikhina[1], Valery P. Glazunov[2], Tatyana S. Zaporozhets[1], Mikhail Y. Shchelkanov[1] and Irina M. Yermak[2,*]

[1] G.P. Somov Institute of Epidemiology and Microbiology, Rospotrebnadzor, 690087 Vladivostok, Russia; krylovanatalya@gmail.com (N.V.K.); olga_iun@inbox.ru (O.V.I.); pott_a.b@mail.ru (A.B.P.); niiem_vl@mail.ru (T.S.Z.); adorob@mail.ru (M.Y.S.)
[2] G.B. Elyakov Pacific Institute of Bioorganic Chemistry, Far-Eastern Branch of the Russian Academy of Science, 690022 Vladivostok, Russia; kravchenko_89@mail.ru (A.O.K.); galin56@mail.ru (G.N.L.); mischenkonp@mail.ru (N.P.M.); glazunov@piboc.dvo.ru (V.P.G.); imyer@mail.ru (I.M.Y.)
* Correspondence: krylovanatalya@gmail.com (N.V.K.); imyer@mail.ru (I.M.Y.); Tel.: +7-9084-486-423 (N.V.K.); +7-9147-005-530 (I.M.Y.)

Table S1. Contacts of CRG tetrasaccharides with the human ACE2 domain according to molecular docking data

CRG	Ligand	Receptor	Interaction	Distance	E (kcal/mol)
κ-CRG	O1 14	O	ALA 387 (A)	H-donor	2.97 -1.9
	O2 58	OD1	ASP 38 (A)	H-donor	2.90 -2.7
	O6 82	O	HIS 34 (A)	H-donor	3.14 -0.7
	O2 16	NH2	ARG 393 (A)	H-acceptor	3.14 -1.8
	O4 37	NZ	LYS 353 (A)	H-acceptor	3.56 -0.8
	O8 43	NZ	LYS 353 (A)	H-acceptor	2.91 -12.6
	O2 58	NZ	LYS 353 (A)	H-acceptor	2.96 -3.7
	O3 60	NZ	LYS 353 (A)	H-acceptor	3.24 -6.8
	O7 85	NZ	LYS 31 (A)	H-acceptor	4.02 -0.8
	O9 87	NZ	LYS 31 (A)	H-acceptor	2.89 -11.4
	O8 43	NZ	LYS 353 (A)	ionic	2.91 -5.1
	O9 87	NZ	LYS 31 (A)	ionic	2.89 -5.2
	C2 65	5-ring	HIS 34 (A)	H-pi	4.35 -0.6
β-CRG	C5 9	OE1	GLU 37 (A)	H-donor	3.46 -0.9
	O2 34	OE1	GLU 37 (A)	H-donor	2.82 -1.9
	O2 55	OD1	ASP 38 (A)	H-donor	3.14 -1.4
	O5 20	NH2	ARG 393 (A)	H-acceptor	3.46 -1.2
	O2 55	NZ	LYS 353 (A)	H-acceptor	3.01 -7.5
	O3 75	NZ	LYS 31 (A)	H-acceptor	3.14 -2.9
	O4 77	NZ	LYS 31 (A)	H-acceptor	3.08 -7.3
λ-CRG	C 4	ND1	HIS 34 (A)	H-donor	3.53 -0.7
	O 55	OD1	ASP 38 (A)	H-donor	2.78 -2.5
	O 55	NZ	LYS 353 (A)	H-acceptor	2.95 -5.2
	O 69	CB	ASN 33 (A)	H-acceptor	3.70 -0.9
	O 69	CA	PRO 389 (A)	H-acceptor	3.52 -0.9
	O 70	CB	ASN 33 (A)	H-acceptor	3.58 -0.8
	O 71	CA	PRO 389 (A)	H-acceptor	3.51 -1.0
	O 71	NH2	ARG 393 (A)	H-acceptor	3.34 -3.0
	O 75	CA	LYS 353 (A)	H-acceptor	3.22 -0.7
	O 77	CB	ALA 386 (A)	H-acceptor	3.59 -0.6
	O 78	NE2	HIS 34 (A)	H-acceptor	3.17 -1.7
	O 86	NZ	LYS 353 (A)	H-acceptor	2.92 -12.3
	O 87	NH2	ARG 393 (A)	H-acceptor	3.28 -1.0
	O 97	NE2	HIS 34 (A)	H-acceptor	3.13 -1.5
	O 69	NH2	ARG 393 (A)	ionic	3.34 -2.6
	O 71	NH2	ARG 393 (A)	ionic	3.34 -2.6
	O 76	NH1	ARG 393 (A)	ionic	3.18 -3.4
	O 76	NH2	ARG 393 (A)	ionic	3.50 -1.9
	O 86	NZ	LYS 353 (A)	ionic	2.92 -5.0

Table S2. Contacts of CRG tetrasaccharides with the SARS-CoV-2 Delta variant RBD according to molecular docking data

CRG	Ligand	Receptor	Interaction	Distance	E (kcal/mol)
κ-CRG	O3 18	N	GLY 496	H-acceptor	3.07 -0.9
	O2 34	OH	TYR 505	H-acceptor	3.02 -1.4
	O2 76	NH2	ARG 403	H-acceptor	2.90 -3.6
	O7 85	NH2	ARG 408	H-acceptor	2.98 -5.7
	O7 85	NH2	ARG 408	ionic	2.98 -4.6
	O9 87	NH2	ARG 408	ionic	3.35 -2.5
β-CRG	O4 77	OD2	ASP 405	H-donor	2.90 -3.6
	O5 59	NH1	ARG 403	H-acceptor	3.08 -1.7
	O6 80	NH2	ARG 408	H-acceptor	2.98 -3.5
λ-CRG	O 53	OE2	GLU 406	H-donor	2.79 -3.9
	O 75	NH1	ARG 403	H-acceptor	3.09 -5.5
	O 77	N	GLY 496	H-acceptor	3.25 -2.3
	O 80	NH2	ARG 408	H-acceptor	2.96 -5.9
	O 82	OH	TYR 505	H-acceptor	2.78 -3.3
	O 75	NH1	ARG 403	ionic	3.09 -3.9
	O 76	NH1	ARG 403	ionic	3.98 -0.6
	O 78	NH2	ARG 408	ionic	3.43 -2.2
	O 80	NH2	ARG 408	ionic	2.96 -4.7
	O 82	NH1	ARG 403	ionic	3.68 -1.3

Table S3. Anti-SARS-CoV-2 activity of different types of carrageenans

Compounds	CC_{50} (µg/mL)	CPE Inhibition assay		RT-PCR assay		
		IC_{50} (µg/mL)	SI	Ct_{ci}	Ct_{ci} - Ct_i	IC, %
**κ-CRG**	≥2000	61.0 ± 6.3	33.0 ± 3.0	25.7 ± 2.7	8.2 ± 0.9	35.9 ± 3.1
**λ-CRG**	≥2000	120.0 ± 13.2	16.7 ± 1.6*	21.2 ± 2.5	3.7 ± 0.4	14.9 ± 1.8*
**Σ-CRG (κ+λ)**	≥2000	85.0 ± 8.3	23.5 ± 2.1*	23.8 ± 2.6	6.3 ± 0.7	26.6 ± 2.2*
**κ/β-CRG**	≥2000	160.0 ± 17.6	12.5 ± 1.5*	20.3 ± 2.4	2.8 ± 0.3	11.0 ± 1.3*
**Ribavirin®**	730 ± 88	207 ± 25	3.5 ± 0.4	19.3 ± 2.5	1.8 ± 0.2	9.7 ± 1.2
**Remdesivir®**	72 ± 9	1.4 ± 0.1	51.4 ± 5.6	28.4 ± 3.4	10.9 ± 1.2	49.5 ± 6.1
**Virus control (DMSO)**				17.5 ± 1.9		
**Cell control (DMSO)**				36.0		

Note: Vero E6 cells were infected with virus (2.0 lg $TCID_{50}$/mL) and simultaneously treated with tested compounds. CC_{50} - 50% cytotoxic concentration; IC_{50} - 50% inhibitory concentration; SI - selectivity indices (CC_{50}/IC_{50}); IC (%) - inhibition coefficient; Ct – the threshold PCR cycle number; Ct_o - threshold PCR cycle number for uninfected cells (cell control); Ct_i - threshold PCR cycle number for infected cells without the drug (virus control); Ct_{ci} - threshold PCR cycle number for the infected cells treated by the investigated compound. Data represent mean ± SD from three independent experiments. * Significance of the differences between the parameters of κ-CRG compared to CRG polysaccharides (λ-CRG, Σ-CRG (κ+λ), and κ/β-CRG) ($p \leq 0.05$)

Table S4. Anti-SARS-CoV-2 action of the κ-CRG/Ech complex (RT-PCR assay)

Compounds	Pretreatment of virus			Pretreatment of cells			Simultaneous treatment			Treatment of infected cells		
	Ct_{si}	Ct_{si} - Ct_i	IC, %	Ct_{si}	Ct_{si} - Ct_i	IC, %	Ct_{si}	Ct_{si} - Ct_i	IC, %	Ct_{si}	Ct_{si} - Ct_i	IC, %
**κ-CRG**	20.3±2.2	3.4±0.4	13.1±1.6*	26.4±3.0	9.5±1.0	41.1±4.5*	24.2±2.7	7.3±0.8	30.3 ±3.1*	21.2±2.3	4.3±0.5	16.9±2.2
**Ech**	23.8±3.1	6.9±0.8	28.4±3.7*	18.4±2.0	1.5±0.2	5.5±0.6*	20.7±2.2	3.8±0.4	14.7±1.8*	18.1±2.1	1.2±0.3	4.4±0.9*
**κ-CRG/Ech**	27.2±3.3	10.3±1.2	45.3±5.9	22.0±2.8	5.1±0.6	20.3±2.4	26.1±2.9	9.2±1.0	39.7±4.2	21.7±2.4	4.8±0.5	19.0±2.3
**Ribavirin®**	NA	NA	NA	NA	NA	NA	19.3±2.3	2.4±0.3	9.1±1.1	19.8±2.2	2.9±0.3	11.1±1.2
**Remdesivir®**	NA	NA	NA	NA	NA	NA	28.0±3.0	11.1±1.2	49.6±5.4	29.2±3.3	12.3±1.2	56.3±6.7
**Virus control**	16.9 ± 2.0											
**Cell control**	36.0											

Note: Vero E6 cells were infected with virus (2.0 lg $TCID_{50}$/mL) and treated with tested compounds in various schemes. The results of the RT-PCR assay were evaluated using the IC (%) - inhibition coefficient; Ct – the threshold cycle; Ct_i – average Ct value for infected cells without the drug (virus control); Ctsi – average Ct value for infected samples after treatment with polysaccharides. Data represent mean ± SD from three independent experiments. * Significance of the differences between the parameters of κ-CRG/Ech complex compared to its components (καppa-CRG and Ech) ($p \leq 0.05$).
